# The prisoner as patient - a health services satisfaction survey

**DOI:** 10.1186/1472-6963-9-176

**Published:** 2009-09-28

**Authors:** Johan Håkon Bjørngaard, Åse-Bente Rustad, Ellen Kjelsberg

**Affiliations:** 1 Department of Public Health and General Practice, Norwegian University of Science and Technology, 7465 Trondheim, Norway; 2 Centre for Forensic Psychiatry, Oslo University Hospital, 0407 Oslo, Norway; 3 SINTEF Health Services Research, 7465 Trondheim, Norway

## Abstract

**Background:**

There is evidence for higher morbidity among prison inmates than in the general population. Despite this, patient satisfaction with the prison health services is scarcely investigated. The aim of the present study was to investigate patient satisfaction with prison health services in Norway and to analyze possible patient and service effects.

**Methods:**

The survey took part in 29 prisons in the southern and central part of Norway, representing 62% of the total prison capacity in Norway. A total of 1,150 prison inmates with prison health services experiences completed a satisfaction questionnaire (90% response rate). The patients' satisfaction was measured on a 12-item index. Multilevel analyses were used to analyze both patient and service characteristics as predictors of satisfaction.

**Results:**

The study revealed high levels of dissatisfaction with prison health services. There were substantial differences between services, with between-service-variance accounting for 9% of the total variance. Satisfaction was significantly associated with a senior staff member's evaluation of the health services possessing adequate resources and the quality of drug abuse treatment. At the patient level, satisfaction was significantly associated with older age, frequent consultations and better self-perceived health.

**Conclusion:**

Prison inmates' satisfaction with the health services provided are low compared with patient satisfaction measured in other health areas. The substantial differences observed between services - even when adjusting for several known factors associated with patient satisfaction - indicate a potential for quality improvement.

## Background

User participation has come to play an increasingly important part in the evaluation of our health services. Patient satisfaction surveys have been conducted in a number of different settings [1]. However, patient satisfaction with prison health services are scarcely investigated [2].

Health workers delivering services in correctional settings face a number of unique challenges. High morbidity rates are often encountered in prison populations, with a particularly high prevalence of transmissible diseases and mental disorders [3]. The organization of the health services varies and the loyalty of the health workers may - justly or unjustly - be questioned, depending on administrative lines, economy and funding.

Coffey [4] in a 2006 literature review of service user research in forensic mental health settings during the period 1990-2004, found that both the volume and the breadth of studies exploring service users' views were limited and limiting. Further, the studies demonstrated significant flaws in terms of the conduct, application, and reporting of the research process. And, given the complexity of ethical issues with largely captive populations, they found a striking absence of discussion of ethical problems in forensic mental health research. The reviewer called for a wider range of approaches to assessing service user perspectives in the prison setting, both methodologically and theoretically. The application of quality criteria should be more consistent and applied more rigorously. The author concluded that we still know relatively little of the experiences of people who use forensic mental health services, and may judge available findings as unreliable.

The quality of the different services providing services is of great importance since prisoners can not, as the general population, choose their health service providers. There is a built-in assumption that expressed dissatisfaction is reflecting deficiencies with the services. However, this assumption has been poorly investigated. Structural service characteristics are in this respect of particular interest, since they are amenable to change by health policy. There is some evidence that administrative/structural measures have been associated with satisfaction [5,6], but the research is not conclusive [7]. Furthermore, the organizational contribution to patient satisfaction scores is questionable. In the patient satisfaction literature, there are some studies that have assessed the relative contribution of the service units to overall satisfaction [5,7-13]]. The results from these studies indicate that organizational contributions to patient satisfaction vary considerably. Hence, this issue should be taken into account when analyzing multi-centre studies on patient satisfaction. We are not aware of any studies that contribute multilevel analyses of satisfaction in prison health services.

In a comprehensive review of the patient satisfaction literature, Crow et al. identified several patient characteristics associated with patient satisfaction [1]. Several studies found clear associations between self-evaluated health status and patient satisfaction. In general, the findings in the literature on the impact of socio-economic status on satisfaction are inconclusive. There are no consistent findings about the relationships between satisfaction and patient's gender, but there is a consistent finding in the literature that older adult patients are more satisfied than younger patients. There is some evidence that ethnicity is associated with satisfaction, but the results are not conclusive.

### The aims of the present study

We wanted to address the apparent paucity of scientifically sound patient satisfaction studies carried out in correctional settings by investigating a large prison population about their satisfaction with the health services provided. We chose a properly validity and reliability checked instrument developed and used in a large national survey on mental health service satisfaction [14], thus enabling us to compare user satisfaction in the prison population with that of patients in mental health services. In addition, we collected data from the prisoners about their prisoner status and sentencing and their self-perceived somatic and mental health status. Lastly, we collected information on staffing, specialist services etc. from the primary health service providers themselves. This study addresses the following research questions:

1. To what extent are prisoners as patients satisfied with the health services in prisons?

2. To what extent can the level of satisfaction with the prison health services be attributed to between-services and within-service factors?

3. To what extent is satisfaction with the prison health services associated with individual characteristics such as demographic status, education, self-perceived health, use of illegal drugs and type of imprisonment?

4. To what extent can service characteristics such as staffing level, competence and prison type explain differences in satisfaction levels between services?

## Methods

### The Norwegian prison health services

All prison health services in Norway, both primary and specialist services, have since 1987 been fully integrated into the general health services in the local community (primary health care) and the larger health region (specialist and hospital services) where the prison is situated. The prison health services are funded and run by the health authorities, not the correctional services. Thus, all health workers in our prisons are fully independent of the prison system, both administratively and budget-wise. While large prisons have health workers that work in the prison only, small prisons have part-time health workers that work in the community health services the rest of the time. Prisoners requiring hospitalisation, somatic or psychiatric, will be admitted to general population services. All health regions have medium and high security psychiatric wards where any prisoner with special mental health requirements may be admitted, as well as patients from the general mental health services. There are no forensic hospitals in the country. Serious and violent offenders with psychotic disorders should at the time of sentencing receive a psychiatric treatment order sentence and be diverted from the correctional system.

### Data collection procedures

After the Regional Committee for Medical Research Ethics approval, all prisons in the Central and Southern part of Norway (N = 29) were asked to participate in the study. All prisons consented and the study was carried out during 2007. The participation rates are presented in Table 1.

**Table 1:** Sample selection

	**N**	**%**
**Total number of inmates**	**1,955**	**100**
- Not present at the time of the survey	131	7
**Present at the time of the survey**	**1,824**	**93**
- Participated in the survey at another prison	31	2
- Unable to read/write Norwegian, English or German	93	5
- Reading/writing disabilities	5	0
- Solitary confinement or ill at the day of survey	48	3
- Previously arranged visits (lawyers, doctors/therapists, or family members)	28	1
**Available at the time of the survey**	**1,619**	**83**
- Refused to participate	123	6
- No record of reason for non-participation	42	2
**Respondents**	**1,454**	**74**
- no prison health service experience	304	16
**Study sample**	**1,150**	**59**

In all but one of the 29 prisons, two qualified psychiatric nurses with extensive clinical prison experience contacted the prisoners, either individually in the cell, at mealtime, or at a meeting set up for the specific purpose of conducting the survey. In one small, remote prison the questionnaires were distributed and collected directly by the prison staff.

### The user satisfaction questionnaire

The prisoners' assessment of quality of care were collected using a previously validated satisfaction scale from an instrument originally developed for use in psychiatric settings [14]. This scale seemed to fit our needs for a comprehensive measurement of patient perceptions relating to the process and quality of healthcare delivery in a correctional setting where prisoners frequently have mental health difficulties in addition to various somatic health complaints. The original instrument was developed based on extensive literature review and patient and expert views and has shown evidence for good content validity, construct validity and test-retest reliability. It consists of 11 items, all with high factor loadings on a single factor, supporting the existence of a uni-dimensional measure of outpatient experiences with the health services. The original 11-item scale was slightly modified in order to suit the prison setting. The original wording was maintained as far as possible in order to facilitate comparison with the results from the psychiatric survey. One of the items was excluded as it was limited to asking about reduction in psychiatric symptoms during therapy while two items addressing the help received during treatment for mental and physical afflictions were added. Hence, our scale consisted of 12 items. In line with previous use of the instrument, factor analysis produced a single factor solution of all items included [14]. A sum satisfaction score was calculated and rescaled to a range from 0 (lowest possible satisfaction score) to 100 (highest possible satisfaction score). Corrected item-total correlations exceeded 0.7 for 11 of the items while one item had a value of 0.4, indicating high correlations between each item and the reminder of the scale. Cronbach's alpha exceeded 0.9.

The questionnaire was developed in Norwegian but translated into English and German in order to make participation possible to non-Norwegian speaking prisoners. 90% of the respondents completed the Norwegian, 9% the English, and 1% the German version of the questionnaire.

The results reported by us pertain to primary and specialist health care combined and include both somatic and mental health services. We found it impossible to ask the prisoners to distinguish between the administrative levels of care, since prisoners are often ignorant of these issues.

### Independent variables

Variables known to be associated with patient satisfaction in the literature, such as age, gender, education, self-perceived health, number of visits in the last three months, duration of treatment episode and ethnicity, were included as independent variables at the patient level [1]. Furthermore, variables measuring patients' self-reported use of illegal drugs at admission and incarceration type were included to further adjust for compositional differences across services. Incarceration type was recorded as remand, ordinary prison sentence or preventive detention (indefinite imprisonment of particularly dangerous but sane individuals).

A senior health worker at each primary health service was asked to respond to subjective statements on the adequacy of the staff resources and the service's quality regarding the delivery of somatic, psychiatric and drug abuse treatment (yes = 1, no = 0). Furthermore, each prison's capacity of prisoners per full-time equivalent health service worker, the size of the prison and the type of prison (open, closed or mixed) were included as independent variables at the health service level. Data regarding the study population are summarised in Table 2.

**Table 2:** Descriptive statistics

**Variables**	**N**	**%**	**Mean (SD)**
**Patient level variables**			
Age	1135		35 (11)
Female	57	5	
Male	1096	95	
Duration of treatment episode:			
<1 month	381	38	
1-6 months	375	37	
> 6 months	258	25	
Number of visits in the last three months:			
1 visit	348	32	
2-5 visits	521	48	
6-12 visits	126	12	
> 12 visits	91	8	
Education			
Obligatory/non education	438	39	
Secondary education	498	44	
University/college education	196	17	
First language:			
Norwegian	762	67	
Other European	184	16	
Non-European	191	17	
Imprisonment type:			
Serving sentence	884	77	
Remand prisoner	216	19	
Preventive detention	50	4	
			
**Prison health service level variables**			
Senior staff member's service evaluation:			
Having enough resources	17	61	
Not having enough resources	11	39	
Doing a good job regarding mental health issues	22	76	
Not doing a good job regarding mental health issues	7	24	
Doing a good job regarding drug issues	21	72	
Not doing a good job regarding drug issues	8	28	
Doing a good job regarding somatic health issues	28	97	
Not doing a good job regarding somatic health issues	1	3	
Capacity of prisoners/Full-time staff equivalents	29		42(31)
Capacity of prisoners	29		71(74)
Prison type:			
Open prison	9	31	
Closed prison	15	52	
Mixed open and closed prison	5	17	

### Statistical procedures

The material was divided into two hierarchical levels - prisons and patients - and multi-level regression analysis was performed with the statistical program Stata [15]. The dependent variable (patient satisfaction) was treated as a continuous variable, and linear regression analysis was performed.

The regression intercepts were allowed to vary randomly across prison health services, thus making it possible to estimate the variance attributed to the service level. The intra-class correlation coefficient (ICC) was used as a measure of the degree of agreement between patients who received treatment at the same prison. Multiplied with 100 the ICC can be interpreted as the percentage of the total variance attributable to the prison health service level. We first analysed the variance attributable to differences between prison health services, without any explanatory variables included and adjusted for the independent variables mentioned above (Table 3). Further on, we analysed each independent variable's association with the satisfaction scale and in a full model ('unadjusted' and 'adjusted' columns in Table 3). Differences were deemed significant when p < .05.

**Table 3:** Multilevel regression analysis of patient satisfaction^1 ^with the prison health services in 29 prisons

**Independent variables**	**Unadjusted^2^**	**p**	**Adjusted^2^**	**p**
**Patient level variables**				
Age	.21	.001	.24	.001
Gender (female = 1)	4.66	.301	5.11	.140
Duration of treatment episode:				
1-6 months compared with <1 month	1.09	.482	-.17	.919
> 6 months compared with <1 month	2.34	.192	-1.00	.605
Number of visits in the last three months:				
2-5 visits in the last three months compared with 1 visit	5.59	< .001	4.56	.004
6-12 visits in the last three months compared with 1 visit	9.96	< .001	10.58	< .001
> 12 visits in the last three months compared with 1 visit	11.49	< .001	10.88	< .001
Drug abuse when not incarcerated^3^	-.63	.150	-.02	.964
Sleeping difficulties^3^	-3.89	< .001	-2.94	< .001
Self-evaluated physical health^3^	-2.41	< .001	-1.17	.119
Self-evaluated mental health^3^	-2.70	< .001	-1.33	.047
Secondary education compared with obligatory/none	1.69	.230	.89	.565
University/college education compared with obligatory/none	5.53	.003	2.25	.278
European compared with Norwegian as first language	1.54	.421	1.22	.567
Non-European compared with Norwegian as first language	-.89	.618	1.72	.380
Remand prisoner compared with serving sentence	.24	.890	1.45	.456
Preventive detention compared with serving sentence	.69	.867	2.51	.559
				
**Prison health service level variables**				
Senior staff member's service evaluation:				
Enough resources compared with not	3.49	.268	7.90	.028
Doing a good job regarding drug issues compared with not	7.77	.007	9.08	.013
Doing a good job regarding mental health issues compared with not	.14	.968	-6.47	.098
Capacity of prisoners/Full-time staff equivalents	-.03	.580	-.04	.586
Prison size (capacity of prisoners)	-.02	.214	-.02	.268
Closed prison compared with open prison	-2.57	.472	.53	.909
Mixed compared with open prison	-2.58	.566	-1.90	.718
Constant			31.77	< .001
				
Prison-level variance	43.83^4^	.007	38.77	.037
Patient-level variance	454.92^4^	< .001	396.71	< .001
ICC^5^	9%		9%	
N	1,144(max)		901	

^1 ^Range from 0 to 100 where 100 is the highest possible level of reported satisfaction

^2 ^Unstandardized regression coefficients

^3 ^Range from 0 to 4 where 4 is the highest level of reported difficulties

^4 ^Prison health service variance and patient-level variance estimates in a model without explanatory variables

^5 ^The percent of the total variance attributable to the prison level

## Results

### Participation rates and sample characteristics

The total capacity of the 29 participating prisons was 2,065. This represents 62% of the total prison capacity in Norway at the time (3,346 in 2007). At the time of the survey, a total of 1,955 prisoners resided in the 29 prisons.

A total of 1,619 prisoners were available for participation in the survey of which 123 actively refused to participate (Table 1). In 42 cases the reason for non-participation was not recorded. In the end, 1,454 prison inmates participated in the study - representing 74% of the total prison population (1,955) and 81% of the inmates eligible for inclusion (1,793). Acknowledging that 174 prisoners were not able to participate due to language/reading difficulties, current illness or solitary confinement, or pre-arranged appointments with lawyer/therapist/family, the participation rate increases to 90% (1,454 of 1,619). A total of 304 prison inmates reported that they did not, and had never previously, used any prison health services. Hence, the present study sample has satisfaction information from 1,150 prison inmates and 82% of these had used the health services recently.

Descriptive sample statistics of the prison health service users are presented in Table 2. The prisons had a mean capacity of 71 prisoners (range 13-398) and the services had a mean value of 42 prisoners per full-time-equivalent clinical position (range 14-175). All but one of the service health workers reported adequate quality regarding delivering somatic health services. Hence, this indicator did not show enough variance to be included in the later regression analysis.

In Table 4 the patients' responses on the 12 satisfaction items are presented, together with a global satisfaction item and the distribution on the questions on self-evaluated health and drug abuse. There was a tendency towards low overall satisfaction with the health services, with 41% being 'very or quite dissatisfied' on the global question.

**Table 4:** Number of respondents, mean (standard deviation) and frequencies of item responses

			**Frequency (%)**				
**Items/scale**	**N**	**Mean (SD)**	**0**	**1**	**2**	**3**	**4**
**Patient satisfaction**							
Help with physical afflictions ^1^	1,085	1.56(1.27)	275 (25)	299 (28)	232 (21)	184 (17)	95 (9)
Help with mental afflictions ^1^	933	1.27(1.30)	365 (39)	209 (22)	174 (19)	113 (12)	72 (8)
Outcome - conversation with professional ^1^	995	1.55(1.10)	177 (18)	341 (34)	291 (29)	129 (13)	57 (6)
Overall treatment outcome ^1^	1,072	1.47(1.03)	192 (18)	381 (36)	340 (32)	117 (11)	42 (4)
Enough time for contact/dialogue ^1^	1,078	1.58(1.09)	187 (17)	354 (33)	316 (29)	171 (16)	50(5)
Clinicians' understanding of patient's situation^1^	1,084	1.61(1.17)	230 (21)	275 (25)	324 (30)	193 (18)	62 (6)
Therapy/treatment suitability^1^	1,077	1.36(1.10)	284 (26)	331 (31)	290 (27)	134 (12)	38 (4)
Follow-up actions carried out^1^	1,034	1.59(1.16)	237 (23)	235 (23)	328 (32)	182 (18)	52 (5)
Communication^1^	1,074	2.28(1.20)	108 (10)	168 (16)	289 (27)	331 (31)	178 (17)
Say in treatment package ^1^	1,043	1.35(1.14)	294 (28)	312 (30)	255 (24)	139 (13)	43 (4)
Information about treatment options^1^	1,070	1.30(1.21)	380 (36)	225 (21)	278 (26)	137 (13)	50 (5)
Information about psychological problems ^1^	1,051	1.42(1.18)	307 (29)	246 (23)	297 (28)	151 (14)	50 (5)
Total score^2^	1,144	38.44(22.14)					
Overall satisfaction ^3^	1,131	2.67(1.22)	273 (24)	192 (17)	377 (33)	211 (19)	78 (7)
							
**Self-evaluated health**							
Mental health ^4^	1,143	2.12(1.29)	159 (14)	208 (18)	319 (28)	247 (22)	210 (18)
Physical health ^4^	1,148	2.05(1.12)	100 (9)	248 (22)	442 (39)	210 (18)	148 (13)
Sleeping difficulties ^5^	1,139	2.21(1.38)	203 (18)	123 (11)	301 (26)	254 (22)	258 (23)
Drug abuse when not incarcerated ^5^	1,127	1.36(1.49)	530 (47)	103 (9)	202 (18)	139 (12)	153 (14)

^1 ^0 and 4 represent the worst and best possible patient experiences, respectively.

^2 ^0 = worst possible experience, 100 = best possible experience

^3 ^0 = very dissatisfied, 1 = dissatisfied, 2 = so-and-so, 3 = satisfied and 4 = very satisfied

^4 ^0 = excellent, 1 = very good, 2 = good, 3 = fair and 4 = poor

^5 ^0 = no, 1 = rarely, 2 = yes, sometimes, 3 = yes, often and 4 = yes, all the time

The patients were least satisfied with issues regarding help with mental health afflictions, having a say in the treatment package and information. The patients were on average most satisfied with the communication with the professionals. However, even on this item 53% reported the three lowest levels of satisfaction. The total satisfaction scale had a mean score of 38 and SD of 22.

### Self-reported health

A total of 40% of the patients reported that their mental health was 'not so good' or 'poor', while 31% reported 'not so good' or 'poor' physical health. 53% of the patients reported that they used illegal drugs while not in prison, most of these 'often' or 'all the time'. 82% of the respondents reported that they had current sleep difficulties, most of these 'often' or 'all the time'.

### The result of the multilevel regression analyses

Table 3 shows the results from the multilevel regression analysis. The crude amount of the satisfaction variance that could be attributed to the service level was 9% (p < .01), and this was not substantially affected by adjustment for the independent variables in the full model.

Age was positively associated with satisfaction (p < .01), while the differences between male and female prisoners was not statistically significant (Table 3). The satisfaction differences between patients with different duration of the treatment episode were small and not statistically significant. However, patients with more frequent consultations were clearly more satisfied compared with those with fewer visits in the last three months (p < .01). For instance, patients with more than 12 visits in the last three months had about 11 scale-points higher satisfaction score compared with those with only one visit in the last three months, adjusted for the other variables in the model. There was no statistically significant association between drug abuse when not in prison and the satisfaction scale. Physical, mental and sleeping difficulties were clearly associated with dissatisfaction in the unadjusted models. In the adjusted model, the association between satisfaction and physical health was reduced and no longer statistically significant.

There was a tendency towards higher satisfaction scores among patients with higher education levels in the unadjusted model. When adjusting for other variables, there were no statistically significant differences between the educational groups. Neither having a foreign language as a mother tongue, nor type of sentence was significantly associated with the satisfaction scale.

Structural characteristics such as prison size, capacity of prisoners per full-time-equivalent health care positions or type of prisons were not significantly associated with the scores on the satisfaction scale. There was a statistically significant tendency towards higher satisfaction levels in prisons where the senior service member evaluated the service's competency regarding the treatment of drug abuse as good, both in adjusted and unadjusted models. In the adjusted model, the staff member's evaluation of having adequate resources was also significantly associated with higher satisfaction scores.

## Discussion

This large prison population study showed that patient satisfaction with prison health services in Norway was skewed towards negative experiences. A substantial part of the variance (9%) could be attributed to the prison health services level, leaving 91% as within-service variance.

### Satisfaction with prison health services

The results from satisfaction studies are usually skewed towards high satisfaction scores [1]. This study shows a contradictory finding in this respect, since the results tended to be skewed towards dissatisfaction. The instrument used in this study has previously been used in a nationwide survey on patient satisfaction with outpatient mental health services in Norway [14]. On a 0-100 scale where 100 was the best possible satisfaction level, the mean score in the current prisoner study was 38 (22 = SD), while the outpatients in the previous study had a mean score of 69 (18 = SD). Hence, the prisoners' mean satisfaction score was substantially lower compared with the mental health patients'. Whether the high level of dissatisfaction reflects the prisoners' problematic life situations or genuinely poor health services, or both, remains equivocal. Due to the dearth of prison health satisfaction studies the basis for comparison is weak. Thus, it is difficult to know whether the substantial level of dissatisfaction is a common property among inmates receiving health services.

The concept of satisfaction as such is not clear and satisfaction with services has been suggested to be influenced by factors not amenable to change by the health services themselves [16,17]. However, the high level of prisoner dissatisfaction requires further attention, given the strong evidence of poor health among prisoners in general [3,18].

### The service level's contribution to satisfaction

As prisoners are not able to choose their health services, it is important to evaluate quality differences between prison health services. This study found a substantial organizational contribution to patient satisfaction (9% of the total variance). Studies have indicated small to no organizational contributions to patient satisfaction scores at the levels of hospitals, wards and clinics (0-5%) [5,7-9,11], while the results from studies of mental health treatment teams vary considerably (0-25%) [8,13]. However, it is important to make a distinction between *compositional *and *contextual *explanations for differences in satisfaction scores between prison health services. Contextual explanations call attention to the organizational, cultural, social and physical shared factors within the prisons, while compositional explanations focus on differences in patient characteristics across health services. The between-services variance reported in this study was not particularly affected by adjustment for several known factors associated with patient satisfaction - a result that supports a contextual explanation.

The health service's ratio of the capacity of prisoners per full-time staff equivalents was not substantially associated with the satisfaction scale, neither was the size of the prison. However, patients in services where the senior staff members evaluated the resource situation as acceptable were more satisfied than patients in other services. However, since this is a cross sectional survey, the question remains open whether it is lack of resources that generates poor quality or if other system deficiencies are interpreted as a lack of resources among the staff.

The senior staff member's evaluation of whether the service was delivering adequate services regarding drug issues was clearly associated with satisfaction, while the same question regarding mental health was not - both in adjusted and unadjusted models. However, given that drug problems and mental difficulties are strongly related, it is important to interpret these results with caution. For instance, there was no substantial impact on satisfaction of drug abuse at the patient level. Furthermore, it is likely that these issues are best understood taken together. It is possible that a better coordination of specialized mental health services - including problems regarding dual diagnoses - and prison health services, would be beneficial for many prisoners' health. There is evidence that the psychiatric morbidity among prison inmates is substantially higher than in the general population [19,20], and prisoners' requests for psychiatric services is prominent [21]. The results from this study calls for further development of mental health services among prisoners, including services directed at drug related problems.

### Prisoner characteristics and satisfaction

Unadjusted for other independent variables, both poor mental and physical health status - as well as sleeping difficulties - were associated with dissatisfaction. In the adjusted model, only poor mental health and sleeping difficulties were significantly associated with dissatisfaction. Sleeping difficulties is generally a large health problem for prisoners [22]. Dissatisfaction due to sleeping difficulties may in part be connected with the services' reluctance to prescribe sleep inducing medication. The prescription of potentially dependence-inducing hypnotics/sedatives are highly regulated in Norwegian prisons [18] and this may cause an expectation gap between patients and care providers. Using drugs outside of prison or not did not substantially influence satisfaction ratings - an interesting result given the high prevalence of drug use among prison inmates. However, the question used did not separate between those abusing different types of drugs. It is possible that the health problems and expectations to services of those abusing hard drugs are different compared to those abusing milder drugs like cannabis.

In line with previous satisfaction studies, age was positively associated with satisfaction. As suggested by Crow et al. [1], this may reflect that older patients are more accepting than younger patients, or they may have lower expectations based on prior experiences. Alternatively, older patients may receive better services, for instance due to a more respectful and caring attitude among the providers. We found no substantial differences in satisfaction between female and male patients or between patients with different ethnic origins. Patients with higher education were more satisfied than those with lower education in the unadjusted model, but not in the adjusted. This indicates that the impact of educational differences was mediated by other variables in the model. Duration of treatment episode was not substantially related to satisfaction, while frequency of consultations was clearly associated with satisfaction - suggesting that the patient-provider relationship is more affected by frequency than duration.

### Strength and weaknesses

Given the cross-sectional design of the study, it is important to caution against drawing causal conclusions from the associations demonstrated.

The results were based on a large sample with a high response rate (90%), which is a clear strength of the present study. Low response rates do often represent a major limitation in patient satisfaction studies [23]. The high response rate in the current study may possibly be explained by the personalized administration of the questionnaires. However, 93 inmates were excluded because they were not able to read or write Norwegian, English or German - a selected group that may to some extent have reduced the sample's representativity.

In a comprehensive review from 1999, Sitzia found, with few exceptions, that the authors demonstrated little evidence of awareness and understanding of such crucial instrument properties as validity and reliability [24]. The instrument used in the current study showed high levels of internal consistency and reliability. Furthermore, the results of the present study were based on an instrument with good psychometric properties and previously used in mental health settings [14].

The senior health worker responses have some limitations. The statements were scored with only two values - yes or no - which limited the variation in the scores. Furthermore, it is possible that the responses could have been influenced by the fact that the service's identity was known. This calls for future research investigating service quality in relation to service resources and competence based on independent evaluators.

## Conclusion

There is strong evidence that prisoners as a group are characterized with elevated mental and physical health related morbidity. This study calls attention to several aspects of the quality of health services from the view of the prisoner as patient. At the individual level, the results suggest that special attention should be paid to the prisoners' mental health problems and sleeping difficulties. At the organizational level, the results showed substantial quality differences between services - a result that indicates a potential for quality improvement. As satisfaction scores were affected by the senior staff member's views and not by the actual staffing levels, attention should be paid to the service's own evaluation and perception of their needs.

## Competing interests

The authors declare that they have no competing interests.

## Authors' contributions

JHB, ÅBR and EK designed the study and developed the self-report satisfaction instrument, based on a previous instrument. ÅBR carried out the survey in the prisons. JHB performed the data analysis and drafted the manuscript. All authors read and approved the final manuscript.

## Pre-publication history

The pre-publication history for this paper can be accessed here:


